# Superficial-type endobronchial metastases from colon cancer: A case report

**DOI:** 10.3892/ol.2014.2473

**Published:** 2014-08-21

**Authors:** KOICHI KURISHIMA, HIROAKI SATOH, KATSUNORI KAGOHASHI, KUNIHIKO MIYAZAKI, TOMOHIRO TAMURA, TOSHIHIRO SHIOZAWA, GEN OHARA, MIO KAWAGUCHI, NORIO TAKAYASHIKI, NOBUYUKI HIZAWA

**Affiliations:** 1 Division of Respiratory Medicine, Mito Medical Center, University of Tsukuba, Mito, Ibaraki 310-0015, Japan; 2 Division of Respiratory Medicine, Faculty of Medicine, University of Tsukuba, Tsukuba, Ibaraki 305-8575, Japan; 3 Division of Pathology, Mito Medical Center, University of Tsukuba, Mito, Ibaraki 310-0015, Japan

**Keywords:** colon cancer, pulmonary metastasis, endobronchial metastasis

## Abstract

Certain internal malignancies, including colon cancer, can develop endobronchial metastasis. The present study reports a case of colon cancer with superficial-type endobronchial metastases in a 76-year-old male. Chest computed tomography revealed small masses and infiltrates in each lung, with bilateral hilar lymph node swelling. Superficial endobronchial tumors in each of the bronchi were unexpectedly found by bronchoscopic examination. A biopsy specimen obtained from the endobronchial tumor was diagnosed as colon cancer. Superficial-type endobronchial metastasis from colon cancer is extremely rare, however, such metastasis should be considered for patients who have a history of colon cancer. There should be no hesitation in performing a bronchoscopic biopsy as an additional examination.

## Introduction

Although endobronchial metastasis is rare, colon, kidney and breast cancer are three of the malignancies that can develop endobronchial metastasis (1,2). A range of conditions, including non-malignant tumors, primary lung carcinoma and endobronchial metastasis of carcinoma from extra-pulmonary organs, may form part of the differential diagnosis of endobronchial lesions in the clinic (3–8). Superficial-type endobronchial metastasis arising from extrathoracic tumors is extremely rare and thus, the incidence remains unknown. To prevent bleeding and airway obstruction caused by endobronchial metastasis, irradiation and photodynamic therapy have been proposed, however, a standard therapy has not yet been established due to its rarity and the outcome of this type of endobronchial metastasis from colorectal cancer has not been previously reported. The present study reports the case of a patient with superficial-type endobronchial metastases in the bronchi, which were unexpectedly found by bronchoscopic examination. Written informed consent was obtained from the patient.

## Case report

**Figure 1:**
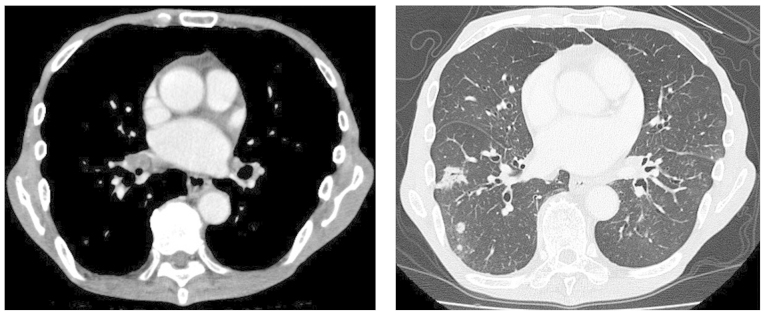
A chest computed tomography scan revealing an ill-defined mass in each lung, with bilateral hilar lymph node swelling.

**Figure 2:**
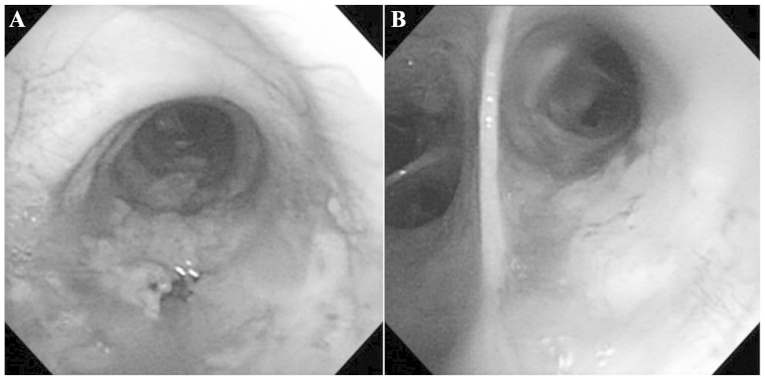
Bronchoscopy images revealing a superficial endobronchial tumor in (A) the left main bronchus and (B) the middle lobe bronchus.

A 76-year-old male was referred to the Mito Medical Center (Mito, Japan) due to a two-month history of vomiting and a loss of body weight. Eight years previously, the patient had undergone a surgical resection for colon cancer. Upon admission, the laboratory examination revealed a hemoglobin level of 12.4 g/dl (normal range, 13–16 g/dl), a hematocrit level of 36.2% (normal range, 40–52%) and a C-reactive protein count of 1.11 g/dl (normal range, ≤0.5 m/dl). The serum level of carcinoembryonic antigen was elevated to 35.1 ng/ml (normal range, ≤5.0 ng/ml). A physical examination revealed right subclavian lymph node swelling, which was pathologically proven to be lymph node metastasis from colon cancer. Computed tomography (CT) of the abdomen revealed multiple metastatic tumors in the liver. X-ray and CT of the chest revealed small masses in the lungs, with bilateral hilar lymph node swelling (Fig. 1). As these pulmonary lesions were observed, bronchoscopy was performed to obtain pathological specimens and to confirm that these lesions were metastatic tumors from colon cancer. Bronchoscopy revealed unexpected superficial endobronchial tumors in the two bronchi (Fig. 2), which were white and marginally elevated flat lesions with irregular margins. No necrotic tissue was identified, however, the development of small blood vessels surrounding the lesions was observed.

**Figure 3:**
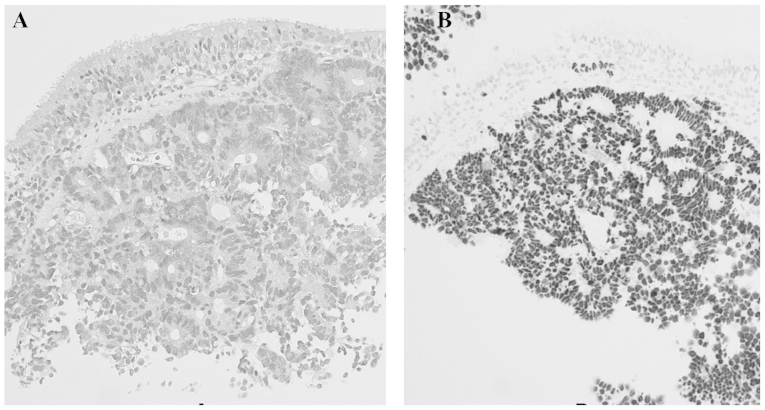
(A) Microscopic features of the endobronchial tumor in the left main bronchus. (B) Immunohistochemical examination revealing positive staining for caudal-type homeobox 2.

Pathological examination of the tumors obtained from the left main bronchus revealed well-differentiated adenocarcinoma with a cribriform pattern, which was consistent with the findings of a resected adrenocortical carcinoma (Fig. 3A). Positive immunochemical staining with caudal-type homeobox transcription factor 2 was confirmed (Fig. 3B). The patient was treated with two courses of TS-1 (80 mg/day for two weeks), but developed multiple brain metastases. One month subsequent to the initiation of the chemotherapy, the patient succumbed to colon cancer. An autopsy was not permitted.

## Discussion

The lung is one of the most common metastatic sites of extra-thoracic tumors, the majority of which are found in the pulmonary parenchyma. Although extremely rare, patients with endobronchial metastasis do occur. The most common symptoms of endobronchial metastasis are coughing and hemoptysis, followed by dyspnea and wheezing. If one or more of these symptoms are present in patients with extra-thoracic tumors, it is possible that such symptoms may be being caused by endobronchial metastasis. However, endobronchial metastasis can also be found in asymptomatic patients. Three of the most common tumors that develop endobronchial metastases are breast, colon and renal tumors (1,2,5–7).

The differential diagnosis of an endobronchial mass lesion includes non-malignant tumors, endobronchial metastasis of carcinoma from extrapulmonary organs and primary lung carcinoma (3–8). Endobronchial lesions can be examined by fiberoptic bronchoscopy, since the majority of lesions are within the view and grasp of the bronchoscopic field. Non-malignant endobronchial tumors usually possess a smooth surface with a uniform color (9). Endoscopic manifestations of an endobronchial metastatic lesion are considerably variable, but polypoid tumors with necrotic material and nodular tumors are commonly observed. Among the primary lung cancer types, squamous cell carcinoma is the most prevalent histological type that is centrally located and exhibits endobronchial extension (4). Polypoid lesions with a necrotic material-covered rough surface are the most frequently occurring tumors in squamous cell carcinoma (10,11). Certain patients with squamous cell or mucoepidermoid lung cancer exhibit superficial or nodular endobronchial tumors (12,13). In addition, although extremely rare, multifocal primary squamous cell carcinomas can develop in each of the bronchi, as observed in the present case (12). The mass-like endobronchial plug (14), which possibly represents an endobronchial infectious process, including mucus plugs distal to a centrally obstructing lesion due to fungus or tuberculosis, also simulates an endobronchial mass on endobronchial examination. In general, it is accepted that without pathological confirmation, the bronchoscopic findings of endobronchial metastasis are not clearly distinct from those of primary lung carcinoma and non-malignant tumors (15,16). In certain cases, the benefits of bronchoscopic examination may be diminished due to the presence of necrotic material interfering with the opportunity to obtain a proper diagnostic specimen (17–19). Therefore, in order to form a proper diagnosis, it is essential to combine the pathological diagnosis with immunohistochemical examination using appropriate specimens obtained by bronchoscopic biopsy.

In primary lung cancer, the endobronchial types have been defined as superficial, nodular and submucosal (20). By contrast, however, there has been no such classification for endobronchial metastatic lesions. If the primary lung cancer classification was to be applied to the present case, the tumor may be classed as of superficial or submucosal type. The endobronchial lesions in the present patient may be the early stage of endobronchial metastasis. These lesions may become enlarged to form nodular lesions, which are usually classified as nodular-type lung cancer.

Endobronchial lesions may form either by invasion from the surrounding tissues, including the lung parenchyma or hilar and/or mediastinal lymph nodes, or by direct seeding within the bronchial wall (1,2). The endobronchial metastasis type in the present patient may be due to direct metastasis to the bronchus or endobronchial invasion of mediastinal or hilar lymphadenopathy. Although endobronchial metastasis is an extremely rare tumor presentation, the possibility that colon cancer can develop superficial endobronchial tumors in each of the bronchi should be considered.
